# Supplementary material for: Proper Functions of Peroxisomes Are Vital for Pathogenesis of Citrus Brown Spot Disease Caused by Alternaria alternata
Source: J Fungi (Basel). 2020 Oct 26;6(4):248. doi: 10.3390/jof6040248 (PMC7712655; doi:10.3390/jof6040248)
Supplement: Supplementary file 1 [file jof-06-00248-s001.pdf]

Table S1. Oligonucleotide primers used in this study

Primer	Sequence (5’→3’)
F0	GTTCCCAGGTGGATGGTTGCG
F2-1	TATCTGATGGCCGGGCTCATG
qPCR1	TTAAGCAGCCTGCGACTACC
qPCR2	TGGACGTATCGTCGGTTTC
R2	TCCTGTGTGAAATTGTTATCCGCTTCTCTTCTTCGTCTGCTCGT
F3	gtcgtgactgggaaaaccctggcgCGCAACAACAACCGAAATCG
R3	AGGCTAACGGACGGATGAAG
R0	GAAATGTTCTAGCCGAAAGCAGGG
hyg3	GGATGCCTCCGCTCGAAGTA
Hyg4	CGTTGCAAGACCTGCCTGAA
M13R	GCGGATAACAATTTCACACAGGA
M13F	GCCAGGGTTTTCCCAGTCACGAC
Pex6_cp1 3.0	AATGCGGCCGCCCATCCATCTCTGCTTCTTTCGGA
Pex6_cp2 pst1	ATCTGCAGTTAAGAGTACAAATCGTCATCCTG
